# Preferences of Information Dissemination on Treatment for Bipolar Disorder: Patient-Centered Focus Group Study

**DOI:** 10.2196/12848

**Published:** 2019-06-25

**Authors:** Berit Kerner, Annette S Crisanti, Jason L DeShaw, Janika-Marie G Ho, Kimmie Jordan, Ronald L Krall, Matt J Kuntz, Aurélien J Mazurie, Anastasiya Nestsiarovich, Douglas J Perkins, Quentin L Schroeter, Alicia N Smith, Mauricio Tohen, Emma Volesky, Yiliang Zhu, Christophe G Lambert

**Affiliations:** 1 Semel Institute, University of California Los Angeles, CA United States; 2 Department of Psychiatry and Behavioral Sciences University of New Mexico Health Sciences Center Albuquerque, NM United States; 3 National Alliance on Mental Illness Montana Helena, MT United States; 4 Bates College Lewiston, ME United States; 5 National Alliance on Mental Illness New Mexico Albuquerque, NM United States; 6 University of Pittsburgh School of Medicine Pittsburgh, PA United States; 7 TwoFoldChange Consulting Bozeman, MT United States; 8 Division of Translational Informatics Department of Internal Medicine University of New Mexico Health Sciences Center Albuquerque, NM United States; 9 Center for Global Health Department of Internal Medicine University of New Mexico Health Sciences Center Albuquerque, NM United States; 10 Division of Epidemiology, Biostatistics, and Preventive Medicine Department of Internal Medicine University of New Mexico Health Sciences Center Albuquerque, NM United States; 11 Division of Translational Informatics, Center for Global Health Department of Internal Medicine University of New Mexico Health Sciences Center Albuquerque, NM United States

**Keywords:** internet, information seeking, psychiatry, bipolar disorder, patient-physician relationship, decision-making, patient education, therapeutics

## Abstract

**Background:**

Patient education has taken center stage in successfully shared decision making between patients and health care providers. However, little is known about how patients with bipolar disorder typically obtain information on their illness and the treatment options available to them.

**Objective:**

This study aimed to obtain the perspectives of patients with bipolar disorder and their family members on the preferred and most effectively used information channels on bipolar disorder and the available treatment options.

**Methods:**

We conducted nine focus groups in Montana, New Mexico, and California, in which we surveyed 84 individuals including patients with bipolar disorder and family members of patients with bipolar disorder. The participants were recruited using National Alliance on Mental Illness mailing lists and websites. Written verbatim responses to semistructured questionnaires were analyzed using summative content analysis based on grounded theory. Two annotators coded and analyzed the data on the sentence or phrase level to create themes. Relationships between demographics and information channel were also examined using the Chi-square and Fisher exact tests.

**Results:**

The focus group participants mentioned a broad range of information channels that were successfully used in the past and could be recommended for future information dissemination. The majority of participants used providers (74%) and internet-based resources (75%) as their main information sources. There was no association between internet use and basic demographics such as age or geographical region of the focus groups. Patients considered time constraints and the fast pace in which an overwhelming amount of information is often presented by the provider as major barriers to successful provider-patient interactions. If Web-based channels were used, the participants perceived information obtained through Web-based channels as more helpful than information received in the provider’s office (*P*<.05).

**Conclusions:**

Web-based resources are increasingly used by patients with bipolar disorder and their family members to educate themselves about the disease and its treatment. Although provider-patient interactions are frequently perceived to be burdened with time constraints, Web-based information sources are considered reliable and helpful. Future research should explore how high-quality websites could be used to empower patients and improve provider-patient interactions with the goal of enhancing shared decision making between patients and providers.

## Introduction

The importance of informing patients about their disease and involving them in decisions about their treatment has been increasingly recognized in studies on recovery from severe mental illness [1-3]. The claim for shared decision making has been justified on ethical grounds, framed as a basic right to self-determination [4], but an association with positive clinical outcomes has also been reported in some studies [5-9]. Patients with depression described better self-management and reduction of symptoms [5], and patients with schizophrenia experienced faster social recovery, more satisfaction with treatment, and fewer rehospitalizations with such an approach [6,7]. Patients with substance use disorder testified to a higher quality of life, increased decision making ability with regard to drug use, and a reduction in psychiatric symptoms after discharge [8,9]. To our knowledge, unfortunately, there are no such studies on patients with bipolar disorder. Systematic reviews of the literature found some overall evidence for more favorable adherence to therapeutic decisions made by shared decision making [10], but the lack of standardization and insufficiencies in both quantity and quality of the studies have hindered establishment of final conclusions [11]. In the process of shared decision making, patient education is crucial to achieve successful treatment outcomes [12,13].

For patients with bipolar disorder and their family members, the process of seeking information about the disorder and treatment is often challenging. First, multiple, and sometimes contradicting, guidelines are available in the scientific literature and on the internet [14-20]. In addition, complex technical language and frequent updates to the treatment guidelines make it challenging to keep up with the field. Furthermore, the evidence of the best available treatment for each patient may be limited. Some medications could also have undesirable side effects, which, from the patient’s perspective, may outweigh the benefits. Uncertainty about treatment options and benefits could influence the communication processes between doctors and patients and challenge treatment adherence.

The literature is sparse on publications that address information seeking by patients with bipolar disorder [21]. Therefore, we aimed to fill this knowledge gap. We conducted focus groups with patients diagnosed with bipolar disorder and some family members of the patients to gain insight into the process of information seeking and shared decision making. We inquired about how evidence on the comparative effectiveness of treatments could best be communicated to patients and their families to empower them as partners in care and to improve outcomes. The key objectives of this exploratory focus group study were three-fold: (1) to better understand how patients and their family members would prefer to be informed about their disease and the available evidence-based treatment options, (2) to identify facilitators and barriers in the education and information-seeking process, and (3) to test if the perception of usefulness of information obtained was independent of the information channels used.

## Methods

### Study Participants and Research Team

To achieve the study goals, we conducted nine focus groups with 4-12 participants each. Study participants were recruited through the National Alliance on Mental Illness (NAMI) mailing lists in three regions of the United States: Great Falls and Helena, Montana; Albuquerque, New Mexico; and Los Angeles, California. Purposive sampling was used to select participants who had the potential to provide rich, relevant, and diverse perspectives. During the first year of this study, three focus groups of 11-12 participants each were conducted, with participants of all ages. Participants were either individuals with bipolar disorder or family members of individuals with bipolar disorder. During the second year, three focus groups were conducted with a special focus on participants aged 18-24 years, and three focus groups were held with elderly participants, most aged 65 years and older; these focus groups were smaller in size, between 4 and 11 participants each, and restricted to individuals with bipolar disorder. The participants were reimbursed for their participation with a gift certificate for a major retailer, and 100% study retention was achieved. For consistency, the focus groups were led by the principal investigator of the study. In addition, each focus group included 2-3 study members who took notes and at least one clinician whose primary role was to provide clinical support, if needed.

### Setting and Informed Consent

The focus groups took place at four different locations—Great Falls, Montana; Helena, Montana; Albuquerque, New Mexico; and Los Angeles, California—between September 23, 2017, and December 15, 2018. The focus groups lasted for 2-3 hours and were held at local NAMI centers or hotel conference rooms at a convenient time for the participants.

All procedures involving human subjects/patients were approved by the Institutional Review Board of the University of New Mexico (#16-243). All participants provided written informed consent to participate in the focus groups and to use the data for research after the nature and possible consequences of the study were explained. After a brief introduction to the study goals and the research team, the focus group participants had the opportunity to discuss the topic and ask questions. The participants were then asked to share their perspectives through anonymized semistructured questionnaires and to respond to the following open-ended questions: (1) “How can we best communicate information about disease management and therapies to newly diagnosed patients with bipolar disorder?” (2) “Where did you get your information about bipolar disorder management?” (3) “What has worked to provide you the education and information you need?” (4) “What has not worked to provide you the education and information you need?” Each participant was asked to describe several sources of information to elicit the maximum number of resources used.

### Quantitative Data Analysis

Demographics of the focus groups were collected without personal identifiers. The demographics were summarized as total numbers and percentages of the total number of participants. The Chi-square test and Fisher exact test for small numbers were used for statistical testing with an alpha level of .05 (two-tailed).

### Data Coding and Qualitative Data Analysis

We used an inductive approach (open coding) to generate themes for the analysis of the written responses using grounded theory and summative content analysis [22,23]. The Consolidated Criteria for Reporting Qualitative Research Checklist was used to report the methodology and findings [24]. Two data coders analyzed the data independently on the sentence or phrase level, if no sentences were used. They then discussed the codes among themselves to generate themes for the analysis through consensus. Codes that were conceptually related were combined, if appropriate, and linked to the more general, overarching themes. The coding process and generation of the themes were then reviewed by the entire research team. Finally, consensus was reached by discussion and included the following themes and information sources: medical doctors and psychiatrists, peer support, patient advocacy organizations, pamphlets, online or in-person educational classes, websites, social media, videos and film, scientific literature and books, mobile phone apps, family members, and social workers and counselors. In some geographical regions, nurse practitioners or therapists served in the function of doctors and psychiatrists. In these cases, the responses were merged with the category “medical doctors and psychiatrists.” The overarching themes and the verbatim phrases that were related to the themes can be found in Table 1.

After the final draft of the paper was completed, feedback on the research findings was obtained from our patient partner advisory group to add validity to our interpretation and ensure that the participants’ own meanings and perspectives were represented and not distorted by the researchers’ agenda and knowledge. The data were then summarized and reported as the total number of times a theme was mentioned and as a percentage of all possible times a theme could have been mentioned. If a participant mentioned several similar or conceptually related information channels, they were counted as one. To better understand facilitators and barriers to the information-seeking process, we further examined the verbatim answers of the focus group participants that referred to this issue. We attempted to group the responses according to a widely accepted model of successful communication between doctors and patients [25,26].

Complete information was obtained on demographic data for age, gender, ethnicity, and race. For the rest of the variables or themes, the theme was annotated as present if mentioned or as absent if not mentioned. Therefore, no missing values were encountered.

**Table 1:** Themes and concepts.

Themes and concepts	Verbatim phrases
Medical doctors and psychiatrists	Therapist, hospitals, Kaiser, Department of Mental Health, outpatient treatment facilities, mental health treatment facilities, clinics, treatment team, provider, mental health treatment community, pharmacist, primary care doctors, hospital staff, emergency room, public services, naturopathic doctors, medical professionals, neurologist, Shodair children’s hospital, medication manager, psychotherapist, nurse, VA^a^ Psychology, behavioral health, inpatient, outpatient, residential program, Veterans administration, nurse practitioner, rehabilitation in mental health facilities
Peer support	Kaiser groups, BP^b^ groups, groups, other people who have mental health issues, peer ed^c^ programs, peer movement, the recovery community, The Sisterhood support group, one-on-one peer advantage, mentor, peer to peer networking, Peer-Bridging (MHC^d^), sharing with others and learning their experiences, talking to others with lived experience, the experience of living with someone with BP, conversations with diagnosed individuals, hearing lived experiences of others, sharing my story, lived experience, 12-step groups, support network, friends who have also struggled, friends with bipolar disorder, recovery international, vet^e^ to vet, talking to others with the same diagnosis
Patient advocacy organizations	NAMI, advocate, Psychosocial Rehabilitation Association of New Mexico (PSRANM), NAMI^f^ conferences﻿, Depression and Bipolar Support Alliance (DBSA), interNational Association of Peer Supporters (iNAPS), Mental Health America, Bipolar/depression alliance
Pamphlets	Hand-outs, booklets, release packets, reading material, forms, print-outs, info that comes with prescriptions, brochure, drug warning pamphlet
Classes in person/online	MFCT^g^ master’s program, seminars, conferences, family to family classes, peer to peer class, provider class, hand-on activities, trainings, power point, educators, college, high schools, patient orientation to educate on meds and diagnosis, charts, workshops, presentations, CEU’s^h^ for license, group education settings, universities, AWARE^i^
Videos and film	Film with information about pros and cons of med, film with interviews with successfully treated patients, YouTube videos, movies, TV shows, documentaries of live experience
Social media	Twitter, facebook, message boards, Yahoo groups, instagram, celebrities who have it, Demi Lovato
Scientific literature and books	Books, scientific papers, articles, B(ehavioral)H(ealth) magazine, journals, medical journals, medical conferences, physician conferences, Psychology today, B(i)P(olar) magazine, publication of synopses in widely distributed magazines, library books, medical journal articles, biographies, memoirs, “An unquiet mind,” “The bipolar survival guide,” public library
Websites	Dr Google, online, online resources, databases, webMD, bipolaradvantage.com, internet, websites dedicated to providing information on medication and medical history, self-paced web course, Mental Health resources online, NIMH^j^ websites, NCBI^k^, Google Scholar, Google, The Wellness Recovery Action Plan (WRAP), do your own research online, blogs, articles on internet, University of New Mexico (UNM) psychiatry website, NAMI websites, internet research, research online, drugs.com, beyond meds website, mad in America website
Mobile phone apps	B(ipolar)D(disorder) app
Family members	cousin, son, dad, father
Social workers and counselors	counseling, caseworkers, case management

^a^VA: veterans affairs.

^b^BP: bipolar disorder.

^c^Ed: education.

^d^MHC: Mental Health Court.

^e^Vet: veteran.

^f^NAMI: National Alliance on Mental Illness.

^g^MFCT: Marriage, Family, Child Therapist.

^h^CEU: Continuing Education Unit.

^i^AWARE: Arming Women Against Rape and Endangerment.

^j^NIMH: National Institute of Mental Health.

^k^NCBI: National Center for Biotechnology Information.

## Results

### Demographics

A total of 84 people participated in the nine focus groups. The median age of the participants was 48.5 years, with a range of 18-79 years. Because we oversampled the younger and older age groups during the second year, one-third of the sample belonged to the age group of 18-29 years (n=28, 33%), and almost one-third of the sample was aged 70 years and above (n=23, 27%; Table 2). About two-thirds of the participants were female (n=54, 64%) and almost two-thirds were non-Hispanic white individuals (n=53, 63%). The majority of the participants (n=78, 93%) were diagnosed with bipolar disorder, and only a small number of participants (n=6, 7%) were family members of patients with bipolar disorder but had not been diagnosed with the disease themselves.

**Table 2:** Demographics of the participants in the focus groups (N=84).

Demographics		Values, n (%)
**Age (years)**		
	18-29	28 (33)
	30-39	4 (5)
	40-49	8 (10)
	50-59	5 (6)
	60-69	16 (19)
	≥70	23 (27)
**Gender**		
	Female	54 (64)
	Male	29 (35)
	Other	1 (1)
**Race/Ethnicity**		
	White (non-Hispanic)	53 (63)
	White (Hispanic)	1 (1)
	Black	14 (17)
	Multiracial	10 (12)
	Other	6 (7)

### Channels of Information Dissemination

Overall, the participants mentioned a broad range of preferred channels through which patients newly diagnosed with bipolar disorder could be informed about the disease characteristics and available treatment options (Table 3). Medical doctors and psychiatrists were the most common sources of information (n=38, 45%), followed by peers and patient advocacy groups (n=29, 35% each). Short written material such as pamphlets and in-person or online educational classes were also popular (n=13 each, 15% each). About 15% of participants mentioned that they would prefer to use websites (n=13, 15%) or social media (n=12, 14%) as a source of information. Videos/films were mentioned by 12% of the participants (n=10) and scientific literature/books, by 10% of participants (n=8). Other information channels including mobile phone apps, family members, or social workers/counselors were rarely mentioned (n=2 in each category, 2%).

To better understand preferences for distribution of information, we also asked the focus group participants about information channels that they had used in the past. Again, the answers covered a wide range of information sources. Leading the list were websites (n=62, 74%) and medical doctors and psychiatrists (n=61, 73%), followed by patient advocacy organizations (n=30, 36%) and peer support (n=28, 33%). The scientific literature (n=19, 23%) and family members (n=10, 12%) were also mentioned by some participants. Educational classes and social workers/counselors (n=8, 10% each) were less frequently used. Film/videos (n=3, 4%) and social media (n=2, 2%) were rarely mentioned. Disease-specific mobile phone apps were not used at all (n=0, 0%).

**Table 3:** Frequency of variables in the data set.

Variable		Frequency, n (%)
**Preferred information sources**		
	Medical doctors and psychiatrists	38 (45)
	Peer support	29 (35)
	Patient advocacy organizations	29 (35)
	Pamphlets	13 (15)
	Classes in person/online	13 (15)
	Websites	13 (15)
	Social media	12 (14)
	Videos/film	10 (12)
	Scientific literature/books	8 (10)
	Mobile phone apps	2 (2)
	Family members	2 (2)
	Social workers and counselors	2 (2)
**Information sources used in the past**		
	Medical doctors and psychiatrists	61 (73)
	Peer support	28 (33)
	Patient advocacy organizations	30 (36)
	Pamphlets	5 (6)
	Classes in person/online	8 (10)
	Videos	3 (4)
	Social media	2 (2)
	Scientific literature	19 (23)
	Websites	62 (74)
	Mobile phone apps	0 (0)
	Family members	10 (12)
	Social workers and counselors	8 (10)
**Information sources perceived as helpful**		
	Medical doctors and psychiatrists	25 (30)
	Peer support	23 (27)
	Patient advocacy organizations	25 (30)
	Pamphlets	5 (6)
	Classes in person/online	12 (14)
	Websites	27 (32)
	Social media	2 (2)
	Videos/film	2 (2)
	Scientific literature/books	12 (14)
	Mobile phone apps	0 (0)
	Family members	3 (4)
	Social workers and counselors	3 (4)
**Information sources perceived as not helpful**		
	Medical doctors and psychiatrists	34 (40)
	Peer support	1 (1)
	Patient advocacy organizations	0 (0)
	Pamphlets	4 (5)
	Classes in person/online	0 (0)
	Websites	3 (4)
	Social media	0 (0)
	Videos/film	0 (0)
	Scientific literature/books	2 (2)
	Mobile phone apps	0 (0)
	Family members	3 (4)
	Social workers and counselors	0 (0)

In a post hoc analysis, we did not detect any statistically significant association between website use and regional location of the focus group (χ^2^_2_=2.7, *P*=.26). Based on data collected during the second year of the study, in which focus groups were held separately for two different age groups (Group 1: 18-24 years vs Group 2: 65 years and above), we did not detect any statistically significant associations between age groups and Web-based resource use (χ^2^_1_=0.03, *P*=.87).

### Barriers and Facilitators of the Information-Seeking Process

To learn about potential barriers and facilitators in the process of obtaining information on bipolar disorder, we asked the focus group participants about what has particularly worked or not worked for them to obtain the information they needed to manage the disease. Participants who had used Web-based resources as well as some who had not, perceived the information that they had received on websites to be helpful (n=27, 32%). In contrast, only one-third of all participants considered the information that they had received from their doctors and psychiatrist helpful (n=25, 30%; Textbox 1). For other information sources, the discrepancy was less marked. Patients who had used patient advocacy organizations and peer support found these resources generally helpful (n=25, 30%, and n=23, 27%, respectively). Even though used less frequently, classes in-person or online and books or scientific literature (n=12, 14% each) were generally perceived to be helpful. However, information obtained from family members and social workers or counselors were less frequently considered helpful (n=3, 4% each). Only a small number of people found information obtained through short written materials (n=5, 6%), videos/film, and social media (n=2, 2% each) helpful. Since disease-specific mobile phone apps were not used, they were also not perceived to be helpful. 

When asked about barriers in the process of obtaining information on bipolar disorder, patients mentioned four main issues (Textbox 1). About 40% of focus group participants (n=34, 40%) perceived the information that they had received from their doctors as not always helpful. Unhelpful interactions with family members (n=3, 4%) and peers (n=1; 1%) were also mentioned by some participants. A few participants found that the way in which information was presented in pamphlets (n=4, 4%), on websites (n=3, 4%), or in the scientific literature (n=2, 2%) was not helpful to them or that they had encountered barriers to understanding the information when they used these channels. Other information channels, including patient advocacy organizations and educational classes, were generally seen in a positive light.

When asked about what had worked and had not worked to provide them with education and information needed, the focus group participants most often described aspects of successful or unsuccessful communication between doctors and patients. The responses could be grouped in three categories: introducing choice; describing options, often by integrating the use of patient decision support; and helping patients explore preferences and make decisions (Textbox 1) [25,26]. Some patients testified to a successful patient-doctor relationship that had met their needs and expectations and to effective ways of receiving information in the doctor-patient relationship. However, many other responses pointed to shortcomings in patient-doctor communication, especially with regard to presenting the information known; discussing benefits, risks, and costs; and clarifying the patient’s understanding. Patients also testified that, in their experience, their values and preferences had not been considered; they experienced a more clinician-led doctor-patient relationship as they struggled to be seen as competent and equal partners in decision-making situations. Potentially related to barriers in the communication processes between doctors and patients was the perception by some focus group participants that information provided by the doctor was not trustworthy or helpful. Generally, the focus group participants expressed a sense of responsibility to find the information that was needed to make decisions on their own.

In contrast, information obtained on the internet was described as reliable and useful. When we compared the perceived helpfulness of information obtained from a doctor and that obtained from the internet, the perception of helpfulness was not independent of the source (Fisher exact test, *P*<.05). Only three focus group participants voiced some concern about the potentially overwhelming task to sort through Web-based information. There was no significant relationship between age group and perceived helpfulness of Web-based resources (χ^2^_1_=0.2, *P*=.65).

**Barriers and facilitators of information seeking in the doctor-patient relationship. Representative quotes are provided from focus group participants at the three locations.**

**Introducing choice**

Positive experiences:

- *I can't say that there is anything that specifically hasn't worked. I have benefited from everything.* [Participant from Los Angeles, California]
- *I think treatment has really worked.* [Participant from Los Angeles, California]
- *My providers have worked to provide me with education.* [Participant from Great Falls, Montana]
- *Doctor interactions have worked- keeping me informed.* [Participant from Great Falls, Montana]

Negative experiences:

- *Having choices forced upon me by psychiatrists.* [Participant from Great Falls, Montana]
- *Not being my own advocate has not worked. I have to seek the information I want.* [Participant from Albuquerque, New Mexico]
- *Being put on medication I know nothing about has not worked.* [Participant from Great Falls, Montana]
- *Doctors are the worst for educating. They don't have enough time. Doctors should encourage individuals on UNM website & the website should educate about UNM Psych programs, disorders, management of disorders & laws governing mental health.* [Participant from Albuquerque, New Mexico]
- *The mental health system is so overrun- very hard to get appointments with psychiatrists, then used very medical language that was hard to understand.* [Participant from Los Angeles, California]
- *Psychiatrist often don’t brief fully on what the drug might be doing or side effects.* [Participant from Los Angeles, California]
- *Rehabilitation in mental facilities made it hard for me to get the information I needed.* [Participant from Los Angeles, California]
- *I found talking to others with the same diagnosis was initially the best way and then I connected with NAMI. My doctors were not helpful the majority of the time.* [Participant from Los Angeles, California]

**Describing options, often by integrating the use of patient decision support**

Positive experiences:

- *Asking my psychiatrist about medicines and my disorder has help educate me.* [Participant from Los Angeles, California]

Negative experiences:

- *There is such a generalized abundance of information. It is not something that’s much discussed.* [Participant from Los Angeles, California]
- *Not getting the correct information at the places where I expected it.* [Participant from Los Angeles, California]
- *While my psychiatrist has provided some info, I don't really expect him to teach me about bipolar disorder as he seems more focused on prescribing drugs. He just encourages me to go to groups and see my therapist.* [Participant from Los Angeles, California]
- *I found miscellaneous doctors and psychiatrist to be either ill-informed or poor communicators, poor diagnostic skills, too busy, etc. Some that I collaborated with tried very hard to keep patients out of the hospital with meds.* [Participant from Los Angeles, California]
- *...just needing some of the purple pamphlets that are provided at the doctor’s office.* [Participant from Los Angeles, California]
- *The doctors I've seen have never explained anything nor directed me to groups or places to know more.* [Participant from Los Angeles, California]

**Helping patients explore preferences and make decisions**

Positive experiences:

- *Working with my mental health professional team is most effective since the information can be tailored to me and allows me to ask questions.* [Participant from Great Falls, Montana]
- *What has worked is person to person discussion between me and my doctor.* [Participant from Great Falls, Montana]

Negative experiences:

- *Having doctors who have huge patient caseloads that are only going by what they know and have learned opposed to patients personal accounts.* [Participant from Albuquerque, New Mexico]
- *...feeling like a statistic by the doctors not listening, misguided information.* [Participant of from Albuquerque, New Mexico]
- *The differences in thinking.* [Participant from Albuquerque, New Mexico]
- *Provider not listening to me. Telling me what I should be feeling.* [Participant from Albuquerque, New Mexico]
- *Doctors who are dismissive.* [Participant from Albuquerque, New Mexico]
- *Preaching, shaming, pressuring, blaming, “unsolicited” advice, being “should-ed.”* [Participant from Albuquerque, New Mexico]
- *Somebody lecturing me.* [Participant from Albuquerque, New Mexico]
- *Dictating & criticism.* [Participant from Albuquerque, New Mexico]
- *Know-it-Alls!* [Participant from Albuquerque, New Mexico]
- *One-sided opinionated shrink.* [Participant from Albuquerque, New Mexico]

## Discussion

### Principal Results

Many patients with bipolar disorder and family members of patients with bipolar disorder would prefer to be educated by mental health professionals about their disease and the treatment options available to them. In addition, internet-based resources and websites are increasingly used by patients with bipolar disorder to meet their information needs. In this regard, patients with mood disorder do not differ greatly from patients with other complex medical conditions in the primary care setting [27]. Searching the internet for information on medical conditions is a well-accepted approach [28]. For some population subgroups, searching health information on the Web as an initial source of information has become even more common than talking to a doctor [29]. Our study contributes to the small but growing literature on internet use by psychiatric patients to inform themselves about treatment options and medication side effects [30-35]. Educating oneself about the disease and treatment options has been recognized as an important element in the recovery process [36]. In contrast to other studies, we did not find a difference in internet use between younger and elderly patients [37]. However, our study agrees that very few patients seemed to be aware of quality codes for medical websites on the internet. Nevertheless, compared to other studies, fewer patients reported negative experiences with the internet [38]. This might be related to the fact that most participants in our study were involved in peer support groups and patient advocacy organizations, which might have directed their attention to more reliable websites. Overall, disease-specific websites and online courses dedicated to education about psychiatric diseases have become popular, and not only in rural areas, since access to health care professionals has become challenging [39,40].

Although the testimonies of the focus group participants spoke to the frequent use and the perceived usefulness of online resources, they also acknowledged the challenges associated with using the internet. Some focus group participants voiced concerns about the abundant and sometimes conflicting information on bipolar disorder and drug treatment available online. Patients felt that sorting through helpful and unhelpful online information was challenging and sometimes overwhelming. This finding is in accordance with existing research that highlighted concerns about the reliability of health care information on the internet [41]. Information distribution and data use on the internet are challenged by limited oversight or regulation [42]. Ethical issues with internet use have not only concerned patients but also health care providers and other stakeholders [43]. Therefore, creating and monitoring reliable health information websites should be a priority [44].

Despite the widespread use of the internet as an information source, patients and their family members expected to gain most of their knowledge about bipolar disorder and its pharmacological treatment from their health care providers. However, they also admitted that doctors had often not been able to fulfill their expectations. Across all geographical regions, barriers to information gathering were encountered in the doctor-patient relationship. Failed doctor-patient communication was universally perceived as an obstacle to successful treatment outcomes. Our findings are not surprising in light of recent publications and one meta-analysis, which concluded that shared decision making and patient-centeredness of mental health care delivery has not been widely implemented despite strong recommendations [45,46].

To assist in the difficult task of making evidence-based treatment decisions, resources and tools have been developed to aid patients and doctors. Computer-based decision aids have been tested in the research arena [47-49], but the dissemination and implementation of these tools seem to have been limited, as evidenced by the fact that none of our focus group participants reported their use. On the other hand, the focus group participants mentioned that attending conferences, classes, and workshops was beneficial. They also emphasized the importance of peer support, a factor that has been recognized to be central in the recovery process [50,51]. This result supports previous findings that peers could be an important source of information for patients with bipolar disorder [52-55]. Based on this finding, further studies should explore how patients and patient advocates could be involved in the design of digital health interventions and the development of Web-based information sources [56].

### Limitations

Limitations of our study include the relatively small sample size and the heterogeneity of the sample, which was recruited in three states that are culturally very different from each other. Although we saw the opportunity of sampling in underserved and rural areas of the United States as well as in one metropolitan area to elicit a wide range of responses and opinions, we acknowledge that in order to determine statistically significant differences, a larger sample size would be required. In addition, the diagnoses of the patients were not formally verified. Additional information on disease, such as course, duration, and severity was not collected.

Another limitation of our study is related to the sampling among members of patient advocacy organizations, which limits the generalizability of the results. Although this sampling approach was convenient and allowed us to easily reach motivated participants for our focus groups, this approach might have biased the results. Future studies should apply a more broadly defined and unbiased sampling scheme.

Due to the local membership demographics of the patient advocacy organizations in Great Falls, Montana; Helena, Montana; Albuquerque, New Mexico; and Los Angeles, California, certain ethnicities, including but not limited to Hispanic and Asian people, have been underrepresented or completely missing in our sample. Further research should address these shortcomings in a more representative sample.

Our data collection instrument used open-ended questions, and we did not explicitly ask about the use or importance of specific information channels. Consequently, some information sources used in the past might have been missed or forgotten. Our data also do not reflect the influences of Web-based information on the doctor-patient relationship.

### Future Research

Our study highlights a variety of information channels that could be used to inform patients with bipolar disorder about treatment options and choices. Some of these opportunities are currently underused but could potentially be helpful. Given the limited resources in mental health care delivery, directing patients to high-quality websites or utilizing peer support could be beneficial for patients and doctors alike. A recent study suggested that information found on the internet could have both positive and challenging effects on the communication processes between patients and doctors [57]. Therefore, further studies should assess whether Web-based resources could assist doctors and empower patients in shared decision making about health care choices or potentially also cause harm. We would recommend funding agencies to dedicate funding resources to this topic, so that researchers could study the potential of reliable Web-based resources to enhance health care delivery, increase patient satisfaction, and improve outcomes.

**Conclusions**

Web-based resources are increasingly used by patients with bipolar disorder and their family members to educate themselves about the disease and its treatment. Although doctor-patient interactions are frequently perceived to be burdened with time constraints, Web-based information sources are considered a reliable and helpful information source. Future research should explore how high-quality websites could be used to empower patients and improve doctor-patient interactions, with the goal to enhance shared decision making between patients and doctors.

## Abbreviations

AWARE: Arming Women Against Rape and Endangerment
BP: bipolar disorder
CEU: Continuing Education Unit
Ed: education
MHC: Mental Health Court
MFCT: Marriage, Family, Child Therapist
NAMI: National Alliance on Mental Illness
NCBI: National Center for Biotechnology Information
NIMH: National Institute of Mental Health
VA: Veterans Affairs
Vet: veteran
